# Type conversion of secretomes in a 3D TAM2 and HCC cell co-culture system and functional importance of CXCL2 in HCC

**DOI:** 10.1038/srep24558

**Published:** 2016-04-27

**Authors:** Yu Lu, Shan Li, Liping Ma, Yan Li, Xiaolian Zhang, Qiliu Peng, Cuiju Mo, Li Huang, Xue Qin, Yinkun Liu

**Affiliations:** 1 Department of Clinical Laboratory, First Affiliated Hospital of Guangxi Medical University, Nanning 530021, Guangxi, China; 2 Liver Cancer Institute, Zhongshan Hospital, Fudan University, Shanghai 200032, China; 3 Cancer Research Center, Institute of Biomedical Science, Fudan University, Shanghai 200032, China

## Abstract

Macrophages play important roles in the tumor microenvironment, driving cancer progression and metastasis, particularly in hepatocellular carcinoma (HCC). However, few studies have assessed the exact secretome composition in HCC. In the present study, the impact of different phenotype of macrophages on HCC cells was investigated. Alternatively activated macrophages (M2) were found to significantly increase the proliferation, migration, and invasion abilities of SMMC7721 cells (all *P* < 0.05). M2 were then co-cultured with SMMC7721 cells to reconstruct the tumor microenvironment. Conditioned medium from 3D single cultures of M2, SMMC7721 cells, and their co-culture system were analyzed using quantitative proteomics via iTRAQ labeling combined with mass spectrometric analysis. Secretome analysis revealed a total of 159 differential secreted proteins in the co-culture system compared to the single culture systems, with 63 being up-regulated (>1.3-fold) and 96 down-regulated (<0.7-fold). CXCL2 was confirmed to have higher expression in the co-culture system and HCC tissues, and was selected for further investigation. Functional effects data suggested that recombinant human CXCL2 significantly enhanced the migration, invasion ability of SMMC7721 cells, and weakened adhesion ability. While CXCL2 neutralization and CXCR2 blockage significantly inhibited the effects of CXCL2 on SMMC7721 cells, indicating that CXCL2 may play pivotal role in HCC metastasis.

Hepatocellular carcinoma (HCC), the fifth most common cancer in males and ninth in females, accounted for 83% of the total estimated new cancer cases (782,000) worldwide in 2012^1^. Its incidence rates vary by more than 8-fold, with the highest in Eastern and South-Eastern Asia and the lowest in Northern Europe and South-Central Asia (http://globocan.iarc.fr)^1^. Dietary aflatoxin B1 exposure and hepatitis B virus (HBV) infection are known risk factors contributing to such variance^2^. However, according to Peng *et al.*^3^, the morbidity of HCC can be distinct in individuals with the same exposure, indicating that other contributors may exist.

The tumor microenvironment, a ‘complex society’ composed of many cell types along with the extracellular matrix, has been shown to play a pivotal role in driving cancer progression and metastasis^4^. As is well-known, in addition to cancer cells, tumors are composed of stromal cells including fibroblasts, endothelial cells, pericytes, and a variable representation of leukocytes^5^. Among these, macrophages in leukocytes are often the most abundant component^6^, which can represent up to 80% of the cell mass in breast carcinoma^7^. It has been reported that, based on environmental stimuli, macrophages can assume a range of different phenotypes once recruited to tumors^8^. The two extremes of this range obtained *in vitro* are the classically activated macrophage (M1) and alternatively activated macrophage (M2). The former are associated with inflammation and microbial killing activity, whereas the latter are associated with tissue remodeling and angiogenesis^9^.

Of these different activated macrophages, the M2 phenotype has been assigned as the dominating macrophage in cancer, mainly because tumor associated macrophages (TAMs) show a similar molecular and functional profile^10^. Over the years, increasing evidence has indicated that TAMs are active players within the tumor microenvironment in the process of tumor progression and invasion^11^,^12^,^13^. Further, TAMs have been strongly correlated with poor prognosis in different types of solid tumors, including HCC^14^,^15^,^16^,^17^. The possible active mechanism may be the cross-talk between cell–cell and cell–matrix interactions through various cytokines, growth factors, chemokines, and proteases secreted by TAMs. However, few studies have assessed the exact composition of the secretome in such tumor microenvironments and, therefore, the secretory molecules that control tumor progression remain largely unknown. Understanding the cross-talk between HCC-TAM interactions can help elucidate the possible molecular markers for the prediction of HCC outcome as well as of new therapeutic targets.

Herein, the tumor microenvironment was therefore reconstructed through the co-culture of TAMs and HCC cells. Numerous studies have suggested that cells cultured in two-dimensional (2D) cultures lose many important functional characteristics of the cancer tissue phenotype and lack the proper cues found in the tissue microenvironment *in vivo*^18^, while three-dimensional (3D) culture conditions closely resemble *in vivo* tissue culture conditions biochemically and biomechanically^19^,^20^. It has been reported that various cells, including HCC, epithelial, and nucleus pulposus cells, among others, exhibit completely different phenotypes when cultured in monolayers or 3D scaffolds such as matrigel or agarose hydrogels^21^,^22^,^23^. Therefore, to obtain a culture system similar to that of the HCC microenvironment *in vivo*, M2 and HCC cells were cultured in a 3D culture system with agarose hydrogel scaffolds instead of the regular 2D monolayer culture. Quantitative proteomics using iTRAQ labeling combined with mass spectrometry were then used to compare the protein profiles of the co-culture system and those of the single culture systems.

## Results

### M2 single cultured conditional medium (M2CM) could enhance the proliferative, migratory, and invasive capacity of HCC cells

**Figure 1:**
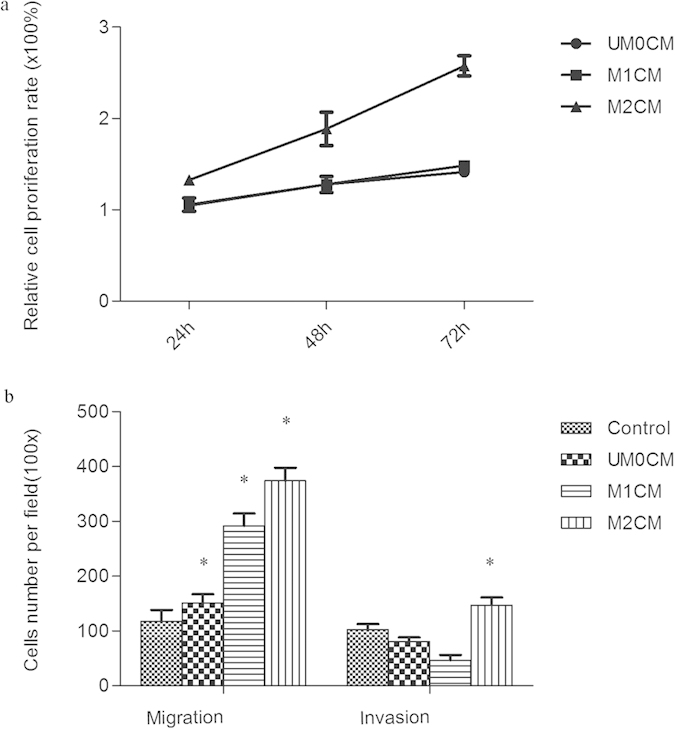
Impact of different activated macrophages (UM0/M1/M2) on SMMC7721 cells. (**a**) CCK8 assay detected the cell proliferation after treated with conditioned medium (CM) of UM0, M1 and M2. Compared with UM0CM and M1CM, M2CM was found to significantly increase the proliferation ability of SMMC7721 cells. (**b**) Influence of UM0CM, M1CM and M2CM on migration and invasion abilities of HCC cells. They were all found to significantly enhance the migration abilities of SMMC7721 cells, but only M2CM significantly increased the invasion abilities of SMMC7721 cells. *p < 0.05 (all compared to the control group and analyzed by Student’s t-test).

M1 and M2 macrophages were further characterized with respect to their influence on tumor cell proliferation, migration, and invasion. Compared with UM0CM and M1CM, M2CM was found to significantly increase the proliferation ability of SMMC7721 cells from the first 24 h of stimulation (all *P* < 0.05), while not much difference was observed between UM0CM and M1CM (Fig. 1a). For the migration assay, the CM of UM0, M1, and M2 were all found to significantly enhance the migration abilities of SMMC7721 cells (all *P* < 0.05; Fig. 1b). The effect was more pronounced in M1CM and M2CM, probably due to both M1 and M2 being activated macrophages and secreting a larger amount of chemoattractants. However, for the *in vitro* invasion assay, the number of invading SMMC7721 cells in the UM0CM, M1CM, M2CM, and control groups was 80.30 ± 8.02, 46.3 ± 9.61, 147.0 ± 14.10, and 102.7 ± 10.21, respectively. Only M2CM was found to significantly increase the matrigel invasion abilities of HCC cells when compared with the control group (*P* = 0.012; Fig. 1b).

**Figure 2:**
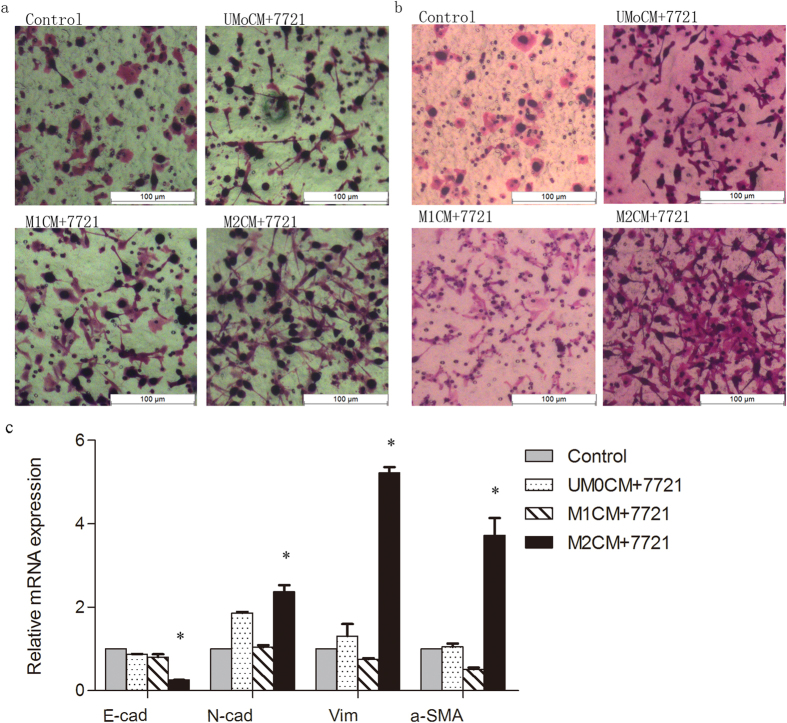
The morphology change of SMMC7721cells during the migration and matrigel invasive assay. (**a,b**) Migration assay and matrigel invasive assay. When treated with M2CM, the morphology of SMMC7721 cells was found to have changed into a diffused fibroblast-like morphology characteristic of EMT, this was not observed with the remaining groups; (**c**) Changes in the mRNA expressions of the EMT-related genes in SMMC7721 cells after treated with UM0CM, M1CM and M2CM. A down-regulation of E-cadherin (E-cad) and up-regulation of N-cadherin (N-cad), vimentin (Vim) and α-smooth muscle actin (α-SMA) were found after M2CM stimulation. *p < 0.05.

Interestingly, when treated with M2CM, the morphology of SMMC7721 cells was found to have changed into a diffused fibroblast-like morphology characteristic of EMT; this was not observed with the remaining groups (Fig. 2a,b and Supplementary Figure S4). Therefore, the common markers of EMT, including E-cadherin, N-cadherin, vimentin, and α-smooth muscle actin (α-SMA), were further investigated using qRT-PCR. Total RNA of SMMC7721 cells treated with M2CM for 48 h were collected and the relative expression of E-cadherin, N-cadherin, vimentin, and α-SMA were detected. Our data showed a down-regulation of the prototypical epithelial cell marker (E-cadherin) and an up-regulation of mesenchymal markers (N-cadherin) after M2CM stimulation; vimentin and α-SMA, expressed in mesenchymal cells and myofibroblasts, respectively, were also found to be increased as mesenchymal markers (Fig. 2c).

### Identifying the alteration of secreted proteins in the 3D co-culture system

**Figure 3:**
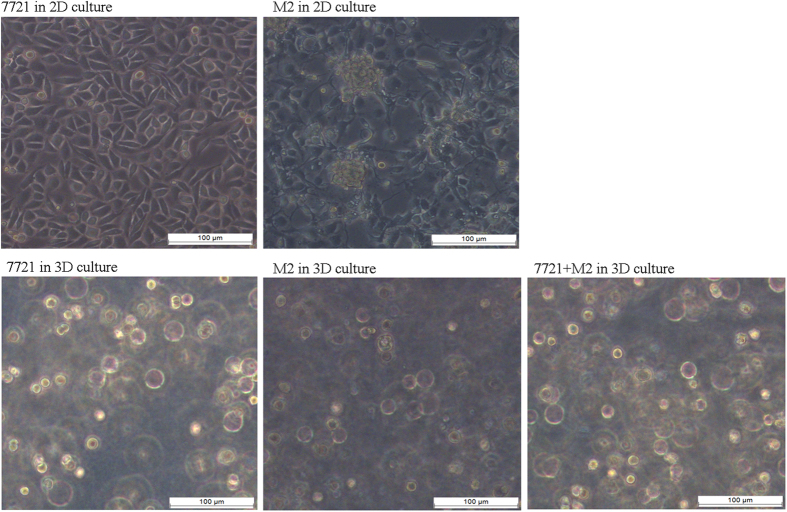
The morphology of SMMC7721 cells, M2, and SMMC7721 + M2 cultured under the three-dimensional condition; morphologies of SMMC7721 cells and M2 in two-dimensional culture condition were also showed.

**Figure 4:**
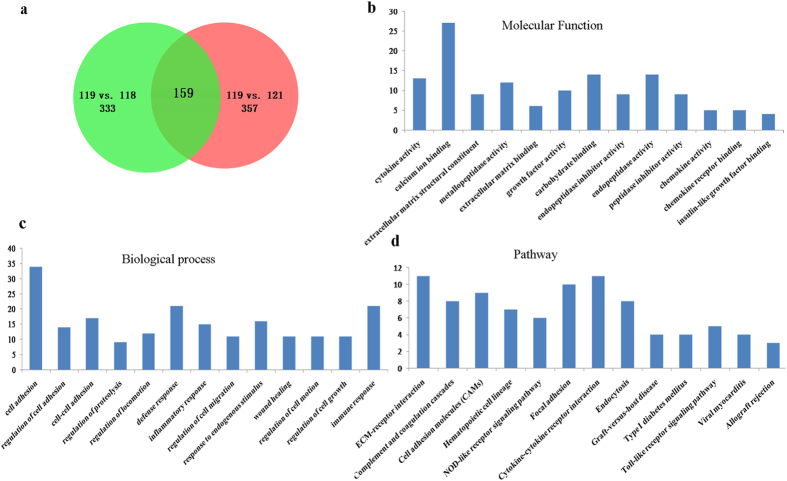
Proteomic identification of the secretome from the conditioned medium (CM) of the different 3D culture systems (SMMC7721, SMMC7721-M2 co-culture, and M2). (**a**) The Venn diagram descript the composition of the differentially expressed proteins among the three culture systems, 118 isobaric tag- M2CM, 119 isobaric tag- co-culture CM, 121 isobaric tag-SMMC7721CM. A total of 595 secretory proteins were identified among the three 3D culture systems, with 333 differentially expressed in the co-culture system when compared with the M2 culture system, and 357 when compared with the SMMC7721 culture system, 159 overlaps were found between them; (**b–d**) Molecular function, biological process and pathway analyses of the differentially expressed proteins.

For 3D co-culture, SMMC7721 cells were cultured with M2 in agarose (Fig. 3). Next, the CM from the different 3D culture systems (SMMC7721, SMMC7721-M2 co-culture, and M2) were collected and 150 μg of the protein was denatured, alkylated, digested, and labeled with the iTRAQ tags. After mixing, they were separated by a strong cation exchange column and analyzed using an RPLC column and QSTAR XL LC/MS/MS system for protein identification and quantification. A total of 595 secretory proteins were unambiguously identified among the three 3D culture systems. Of these, 333 proteins were differentially expressed in the co-culture system when compared with the M2 culture system, while 357 were differentially expressed when compared with the SMMC7721 culture system. When comparing the co-culture to both M2 and SMMC7721 culture systems simultaneously, a total of 159 proteins were found to be differentially expressed in the co-culture system (Fig. 4a), with 63 defined as up-regulated (>1.3-fold; Table S1) and 96 as down-regulated (<0.7-fold; Table S2) according to the cutoff criteria defined above.

### The molecular features of the differentially-expressed proteins

According to the database search result, the differentially-expressed proteins mainly consisted of transporter proteins (12.3%), peptidase (10.5%), transmembrane receptors (7.6%), enzymes (6.4%), and cytokines (5.8%). Following analysis using the DAVID Bioinformatics Resources, these were further classified based on the biological process and molecular function of gene ontology analyses (classification of subcellular distributions was not conducted as all the proteins were secreted; Fig. 4b–d). The results indicated that the molecular function of the differentially-expressed proteins included cytokine activity, calcium ion binding, and extracellular matrix structural constituents, among others; the most common biological process of these proteins was cell adhesion. Furthermore, most of the differential proteins are involved in the ECM-receptor interaction, cell adhesion molecules, and cytokine-cytokine receptor interaction pathways.

### Validation of differentially-expressed proteins

**Figure 5:**
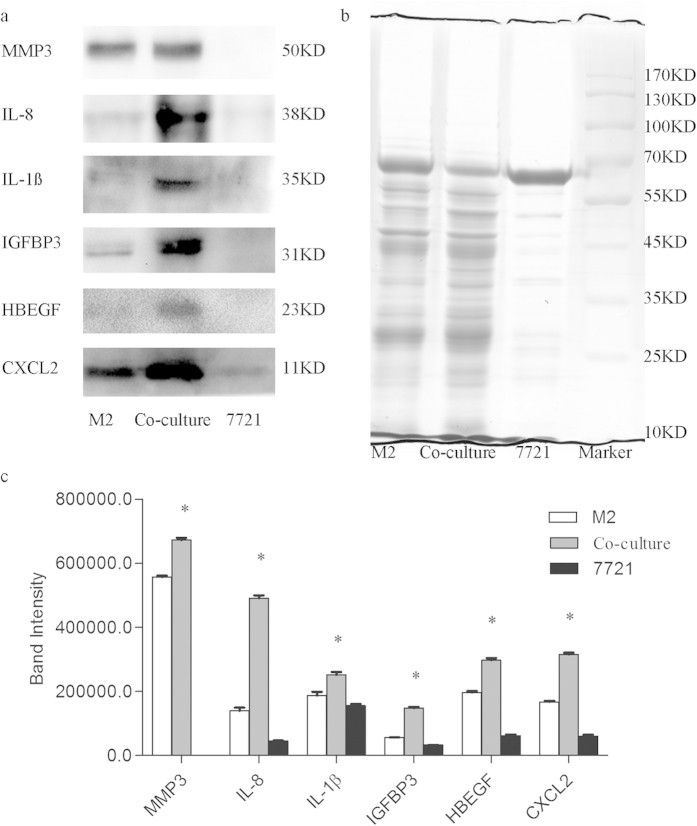
Expression analyses of the secreted proteins. (**a**) Western blot was used to validated the differentially expressed proteins. All six proteins were expressed highest in the co-culture system, four (IL-8, IL-1ß, HBEGF, and IBP3) were completely coincident with the iTRAQ quantification trend, while MMP3 and CXCL2 were found to be more highly expressed in the M2 culture system than in the SMMC7721 culture system. (**b**) Coomassie staining. As all the samples were conditioned medium and β-actin or GAPDH can’t be used as reference to confirm equal protein loading (40 ug), coomassie staining was therefore used; it seems that less protein content of SMMC7721 cells was plotted, as compared to the M2 and Co-culture CM, maybe because the most abundant protein in all CM is albumin (the protein line at 60–70 kD), and CM of 7721 cells contains relative more albumin. (**c**) Bar graph of IL-8, MMP-3, IL-1ß, CXCL2, HBEGF and IBP3 based on densitometric analysis of western blot image and analyzed by Student’s t-test. *p < 0.05.

To confirm whether these proteins were differentially expressed in the 3D co-culture system, western blot was performed selecting six up-regulated (IL-8, MMP-3, IL-1ß, CXCL2, HBEGF, and IBP3) proteins for further verification. The densitometry reading of these proteins and immunoblot bands were analyzed using Quantity One. The results showed that four proteins (IL-8, IL-1ß, HBEGF, and IBP3) were completely coincident with the iTRAQ quantification trend. MMP3 and CXCL2, though not fully consistent with the iTRAQ quantification results, were found to be more highly expressed in the M2 culture system than in the SMMC7721 culture system, and even more so in the co-culture system (Fig. 5).

**Figure 6:**
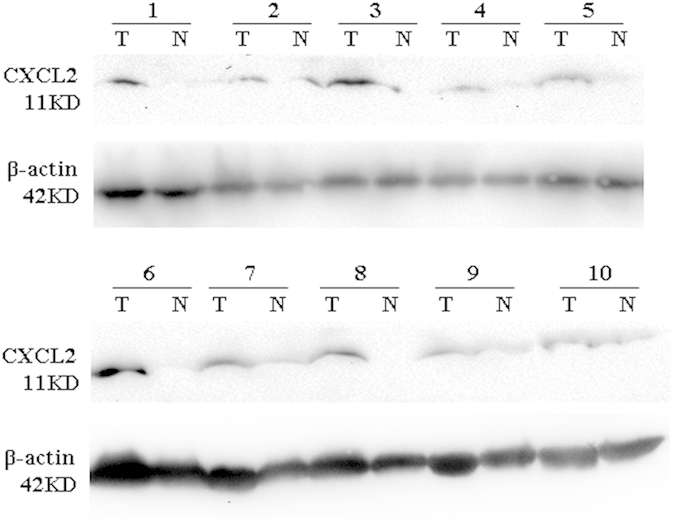
The result of western blot for CXCL2 protein in HCC tissues and corresponding non-tumor normal tissues of HCC patients. The expression CXCL2 was much higher in all HCC tissues compared to the corresponding non-tumor normal tissues. T, tumor tissue; N, corresponding non-tumor normal tissues.

As shown by the iTRAQ quantification results, IL-8 was the most elevated factor in the co-culture system relative to both SMMC7721 cells and M2 cultured alone. Further, its positive role in HCC metastasis by inducing EMT has been clearly demonstrated^24^. Given that CXCL2 is a functional analogue of IL-8 which also significantly increased in the co-culture system, its expression in the human HCC tissue was also assessed. As showed in the western blot result, CXCL2 was found much higher in all HCC tissues compared to the corresponding non-tumor normal tissues (Fig. 6), indicating that CXCL2 may play a similar pivotal role to that of IL-8 in HCC development. Since CXCL2 has been proved to contribute to tumor growth and angiogenesis^25^,^26^, while little is known about whether it is involved in the process of HCC metastasis, a series of experiments focusing on migration and invasion were therefore carried out in the presence of varying levels of recombinant human CXCL2.

### CXCL2 promoted cell migration and invasion but suppressed cell adhesion

**Figure 7:**
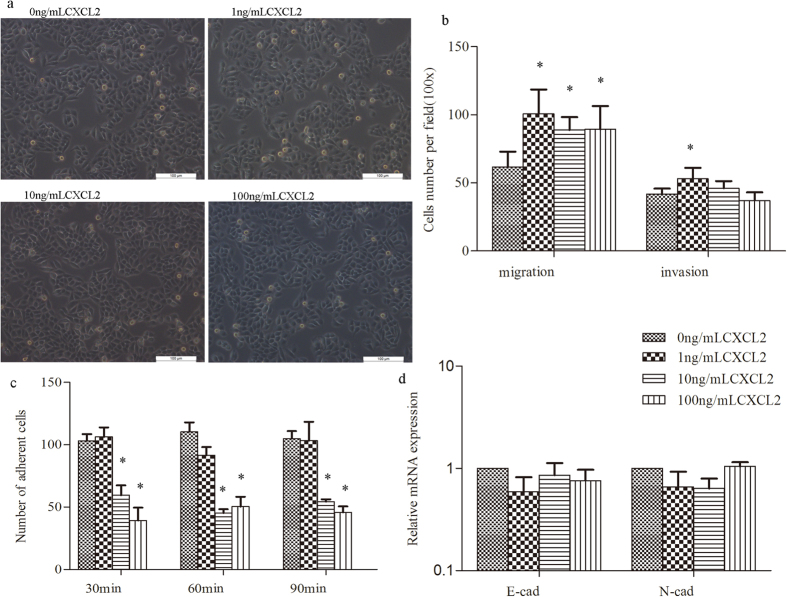
Effect of varying levels of CXCL2 on HCC cells. (**a**) The morphology of SMMC7721 cells after treated with different does of CXCL2, of which didn’t showed much change compared to the untreated cells. (**b**) CXCL2 stimulation could promoted the migration ability of SMMC7721 cells at any chosen concentration, and exerted the most pronounced increase in cell migration with 1 ng/mL CXCL2; however, significantly higher invasion ability was found only when treated with 1ng/ml CXCL2; (**c**) HCC cells exhibited a significantly lower adhesion capacity on FN-coated surfaces after treatment with 10 and 100 ng/mL CXCL2, regardless of the incubation period. (**d**) No difference was found in EMT-related markers after normalized toβ-actin. *p < 0.05.

After treating with different does of CXCL2, the morphology of SMMC7721 cells didn’t showed much change (Fig. 7a). However, those treated cells were found to have significantly increased migration ability as compared to the untreated cells (Fig. 7b). Further, stimulation with 1 ng/mL CXCL2 exerted the most pronounced increase in cell migration. Additionally, HCC cells were found to have significantly enhanced matrigel invasion ability under 1 ng/mL CXCL2 stimulation, while no difference was found with the rest conditions. With regard to the cell adhesion ability, SMMC7721 cells exhibited a significantly lower adhesion capacity on FN-coated surfaces after treatment with 10 and 100 ng/mL CXCL2, regardless of the incubation period. On the other hand, no difference was observed under 1 ng/mL CXCL2 stimulation for any incubation period (Fig. 7c).

Since the cells possessed enhanced invasion but weakened adhesion ability, their expression of invasion-related genes was assessed. Nevertheless, no significant difference was observed when considered the EMT-related markers, under any stimulating condition (Fig. 7d).

### CXCL2 neutralization weaken the migration and invasion ability of HCC cells

**Figure 8:**
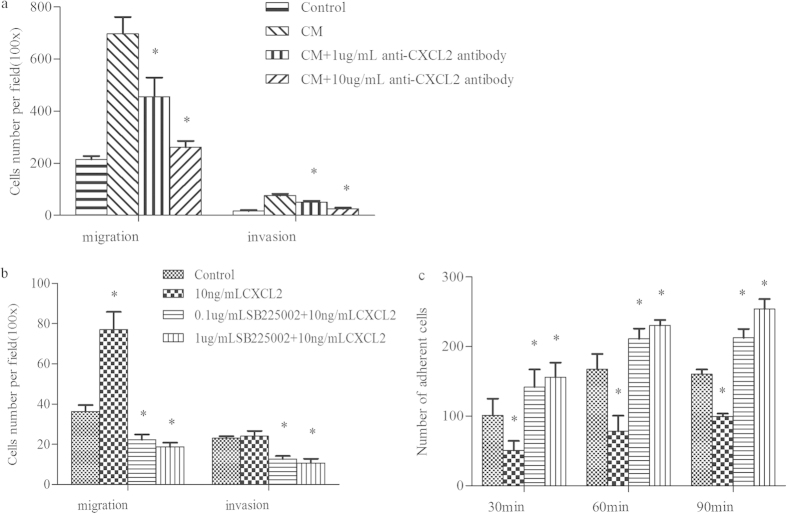
Influence of CXCL2 on SMMC7721 cells after CXCL2 neutralization and CXCR2 blockage. (**a**) Conditioned medium (CM) from the co-culture system could dramatically increased the migration and invasion ability of HCC cells, compared with the normal control; but when co-culture CM was treated with 1 ug/mL or 10 ug/mL anti-CXCL2 antibody, both of these effects were significantly weaken, along with a dose-dependent reduction effect (*compared with the co-culture CM group and p < 0.05). (**b**) SMMC7721 cells showed a significantly decreased cell migration and invasion ability following the treatment with a specific CXCR2 antagonist (SB225002), in both 0.1 ug/ml and 1 ug/ml concentration. (**c**) The adhesion ability of SMMC7721 cells were significantly increased in all 30, 60 and 90 min after treated with 0.1 ug/ml and 1 ug/ml SB225002, comparing to either 10 ng/ml CXCL2 group or blank control group. *p < 0.05.

The pivotal role of CXCL2 was further confirmed when it was neutralized by anti-CXCL2 antibody. As showed in Fig. 8a, compared with the normal control, conditioned medium from the co-culture system could dramatically increased the migration and invasion ability of HCC cells (697.67 ± 64.08 vs. 214.67 ± 12.74 and 75.67 ± 6.43 vs. 16.00 ± 4.00, respectively); However, when co-culture CM was treated with different does of anti-CXCL2 antibody, both of these effects were significantly weaken, no matter in 1 ug/mL or 10 ug/mL concentration, along with a dose-dependent reduction effect.

### CXCR2 blockage inhibited the effects of CXCL2 on SMMC7721 cells

Because it is known that CXCL2 plays its role by binding to CXCR2, we wanted to explore the possible effect of CXCL2 to SMMC7721 after inhibiting the activity of CXCR2. As showed in Fig. 8b,c, SMMC7721 cells showed a significantly decreased cell migration and invasion ability after the blockage of CXCR2, in both 0.1 ug/ml and 1 ug/ml SB225002 concentration, while the adhesion ability were significantly increased in all 30, 60 and 90 min, comparing to either 10 ng/ml CXCL2 group or blank control group.

## Discussion

SeaPrep^®^ Agarose, a polysaccharide with a 17–40 °C gelling temperature and a melting temperature of above 60 °C, is a suitable suspension scaffold for cell culture. It can be used to stimulate and maintain 3D cell culture since agarose hydrogels are porous and exhibit highly biocompatible characteristics and similar mechanical properties as tissues^27^. So far, such scaffolds have been widely used in the 3D culture of various cells, including nucleus pulposus cells, nervous cells, SiHa (human cancer of cervix), and BMG-1 cell lines^23^,^28^,^29^. However, according to Peng *et al.*^30^, who explored the effects of dermatan sulfate-modified agarose gels on neurite extensions in 3D scaffolds, gel stiffness increases with concentration whereas neurite length and gel pore size decrease. Neurite extension was inhibited in dextran sulfate-modified agarose gels at all experimental concentrations (1%, 1.25%, 1.5% and 1.75%) except 0.75% [24]. In the present study, a range of gel concentrations was used to explore the best conditions for 3D co-cultures, and 0.75% was found to be the best.

As is well known, cells in natural multicellular biological systems exist in a complex and dynamic tumor microenvironment, the ‘home’ of tumor initiation and progression; TAMs have been found to play a pivotal role in such an environment. Increasing evidence has indicated that TAMs could promote tumor phenotypes such as angiogenesis, growth, and invasion. The present study demonstrated that M2 macrophages could significantly increase the proliferative, migratory, and matrigel invasive abilities of HCC cells. The above results indicate that the secretomes of M2 macrophages, such as cytokines, growth factors, chemokines, and proteases, may promote the proliferation and invasiveness of cancer cells. To simplify and mimic such an environment, a co-culture model of M2 microphages and SMMC7721 cells in agarose 3D culture systems was developed, and their secretomes were quantified using the QSTAR XL LC/MS/MS system.

In the present study, a total of 159 secreted proteins were found to be differentially expressed in the 3D co-culture system, with 63 of these exhibiting a significantly higher level and 96 being dramatically down-regulated. The results above were compared to the study by Fu *et al.*^24^, who applied an antibody array assay to analyze the supernatant of the non-contact co-culture system with macrophages and HCC cells, and only five cytokines were found to be commonly elevated (IL-8, IL-1ß, IL-6, TNF-α, and CXCL-1). Similar increases in several members of the chemokines superfamily (C-C motif) and the insulin-like growth factor binding protein (IGFBP) family were also noted, but the molecules expressed were not the same (for instance, CCL-4, CCL-20, and IGFBP1 in Fu *et al.*’s results [28], but CCL-3 and IGFBP3 in the present study). The poor overlapping result may be attributed to the fact that i) the HCC cells and macrophages used to co-culture were different (MHCC-97 H and Hep-g2 [28] versus SMMC7721 and UM0 [28] versus M2), and thus exhibited different molecular features; ii) a direct-contact co-culture model was used in the present study, while a non-contact model, which may ignore the cell-cell interaction and lead to a different tumor microenvironment, was used by Fu *et al.* [28]; and iii) the methods applied to acquire the data differed between the studies, and the antibody chip applied by Fu *et al.* [28] consisted of only 80 human cytokines, indicating a relative narrow detection ability.

Indeed, according to the proteomic identification and functional analyses results, the interaction between M2 and SMMC7721 cells induced the alteration of a batch of secretory factors, and among them, some have been suggested to act as growth promoters or metastasis factors in HCC based on previous reports. For example, IL-8, a neutrophil chemoattractant commonly produced in diverse carcinoma cell lines, has been shown to be associated with metastatic potential, angiogenesis, and cell proliferation of HCC^31^, and stimulation from various factors, such as IL-1 and TNF-α, can result in rapid IL-8 transcription and production^32^,^33^. Recent studies also showed that dysfunctional activation of the neurotensin/IL-8 pathway in HCC is associated with increased inflammatory response in the tumor microenvironment, enhanced EMT in cancer, and worse prognosis of HCC patients^34^. TNF-α and IL-6, reported as regulatory cytokines in the tumor microenvironment, have also been revealed as potential prognostic serum biomarkers in early-stage HCC^35^. IL-6, in particular, has been further suggested to be correlated with HCC tumor size and early hepatocarcinogenesis to be dependent on paracrine IL-6 production by Kupffer cells or macrophages^36^,^37^. In sum, the alteration of these secretory proteins may be a consequence of the interaction between M2 macrophages and HCC cells, as well as the cause for M2 macrophage-driven malignancy of HCC cells.

CXCL2, a member of the chemokine superfamily containing a glycine-leucine-arginine motif, is one of the many elevated secreted factors and could be produced by multiple, different cell types, including macrophages and cancer cells^38^,^39^. The abnormal expression of CXCL2 has been observed in tissues of colon cancer patients^40^, plasma of primary chronic lymphocytic leukemia patients, culture supernatants of primary chronic lymphocytic leukemia peripheral blood mononuclear cells^41^, and conditioned medium of prostate stromal cells following stimulation by immortalized prostate epithelial cells^42^. In the present study, CXCL2 was also found to be significantly elevated in the co-culture system of M2 and HCC cells, as well as in tumor tissues as compared to the corresponding non-tumor normal tissues from HCC patients, indicating its possible crucial role in HCC development. A series of experiments were conducted to investigate the involvement of CXCL2 in regulating the metastatic potential of HCC cells; our result indicated that recombinant human CXCL2 could significantly increase the migratory ability of HCC cells compared with untreated cells, in any concentration chosen. This result was consistent with a previous study demonstrating that CXCL2 could provoke a dose-dependent increase of colorectal tumor cell migration *in vitro*^26^. Further, according to Bachmeier *et al.*^43^, CXCL-1 and −2 silencing could down-regulate several metastasis-promoting genes and inhibit the metastatic potential of breast cancer cells. In the present study, HCC cells were also found to exhibit significantly increased invasive ability following stimulation with CXCL2 or co-culture CM. And, after the blockage of its receptor-CXCR2, the effects of CXCL2 to SMMC7721 were inhibited. Further, the similar effects of co-culture CM on HCC cells were also decayed when the containing CXCL2 was neutralized by its specific antibody. Such findings indicated that, CXCL2 could facilitate tumor cell migration and invasion. However, EMT might be not responsible for these effects, since no EMT-related phenomenon was observed here.

Indeed, possible mechanisms regarding CXCL2 promotion of cancer cells metastasis has previously been reported^44^. The IL-1 and NFκB signaling pathways seem to play pivotal roles in these effects, as NFκB has been shown to be involved in the regulation of the metastatic potential of breast cancer cells through CXCL-1 and −2^43^; further, IL-1 was able to stimulate the NFκB signaling pathway as well as to trigger stromal cells to secrete various chemokines including CXCL2^42^. Taking all of the above factors into consideration, future work assessing the exact mechanism of CXCL2 regulation of HCC metastasis is still needed.

In conclusion, alternatively activated macrophages (M2) were found to significantly contribute to the proliferation, migration, and matrigel invasion of SMMC7721 cells. By using quantitative proteomics, obvious changes in the expression profiles of the 3D M2 and SMMC7721 cells co-culture system were observed, with 63 being up-regulated (>1.3-fold) and 96 down-regulated (<0.7-fold). The additional treatment for SMMC7721 cells with recombinant human CXCL2 were found to exhibit a significantly enhanced migration and invasion ability, along with a weakened adhesion rate. And both CXCL2 neutralization and CXCR2 blockage could significantly inhibit the effects of CXCL2 on SMMC7721 cells, indicating that CXCL2 may play pivotal role in HCC metastasis. These findings present new insights into the molecular signatures of the HCC tumor microenvironment and provide a further, novel, potential target for HCC therapy.

## Methods

### Cell culture and induction

Human HCC SMMC7721 cells were cultured in DMEM, human monocytic cell line THP-1 cells were maintained in RPMI 1640, both supplied with 10% fetal bovine serum, 100 U/mL streptomycin, and 100 U/mL penicillin. The cell lines were obtained from the cell bank of Chinese Academy of Sciences (Shanghai, China), and grown in a humidified atmosphere of 5% CO_2_ at 37 °C. To induce different macrophages, 1 × 10^6^/well THP-1 cells were first seeded in 6-well tissue culture dishes and treated with 200 nM/mL phorbol 12-myristate 13-acetate (PMA) for 24 h to induce them into undifferentiated macrophages (UM0). UM0 were then stimulated into M1 and M2 with 1 μg/mL LPS and 20 ng/mL interleukin (IL)-4, 20 ng/mL IL-13 for 3 additional days, respectively. The characteristics of different macrophages were then identified to confirm if they were successful induced, results were showed in Supplementary Figure S1. After the successful induction of UM0, M1, and M2, serum-free RPMI 1640 was added to these cells and maintained for another 48 h, their conditioned medium (CM) was then collected and stored at −20 °C until further use.

### Effect of different phenotype macrophages on HCC cells

#### Cell proliferative assays

Cell proliferation was measured using a Cell Counting Kit-8 (CCK8, Dojindo Laboratories, Japan). Human HCC SMMC7721 cells were seeded at 5 × 10^3^/well in a 96-well plate and allow adhering overnight. Subsequently, the complete medium was removed and the cells were washed with 100 μL phosphate buffered saline (PBS). A solution of CM from each macrophage and serum containing RPMI 1640 at a ratio of 1:1 was added to cells at a mixture volume of 100 μL/well. After incubation for 24, 48, and 72 h, cells were treated with 10 μL CCK8 reagent and incubated for 1 h. The absorbance of each well was measured on a plate reader at 450 nm. HCC cells incubated with only complete medium were used as the negative control and wells with complete medium but without HCC cells as the blank control. Cell proliferation activity was measured according to the equation: Cell proliferation activity = [(experimental absorbance − blank control absorbance)/(negative control absorbance − blank control absorbance)] × 100%.

#### Cell migration and invasion assays

Cell migration and invasion were performed using a Transwell chamber with a polycarbonate membrane containing 8.0-μm pores (Corning Costar, Cambridge, MA). SMMC7721 cells were firstly incubated with serum-free DMEM(control), UM0CM, M1CM and M2CM, respectively. Following 24 hours’ treatment, conditioned medium was collected, cells were resuspended in serum-free medium and plated in the upper chamber coated with (invasion assay) or without (migration assay) matrigel at a concentration of 5 × 10^4^ cells/well. The upper chamber was then inserted into a well of a 24-well plate containing 300 μL serum-containing medium (10%) mixed with 300 μL of the different conditioned medium. After incubation for 24 h, the non-migrating cells in the upper chamber were wiped with cotton swabs and removed, and those that had migrated through the pores were fixed with 4% paraformaldehyde for 30 min and stained with 10% Giemsa for 30 min. HCC cells were cultured in a mixture of 300 μL serum-containing medium with 300 μL serum-free medium for 24 h as a control. The invaded/migrated cells in each chamber were observed at 100x magnification; five random fields were chosen to count and determine the mean number for each group.

### Formation of the tumor microenvironment by the co-culture of M2 and SMMC7721 cells in agarose hydrogels

M2 and SMMC7721 cells were washed in PBS and collected after trypsinization for further 3D culture. Since the different agarose gel concentrations affect mechanical stiffness^30^, 3D cultures were first performed using 5 × 10^6^/mL SMMC7721 cells and 0.5%, 0.75%, 1% and 1.5% agarose in RPMI1640. At the 0.5% concentration, the cells partially grew as adherent monolayers; while at 1% and 1.5% agarose, the CM concentration in these culture systems were lower than that of 0.75% agarose, indicating a relative lesser exchange of various substances. Therefore, 0.75% agarose was chosen as the optimal conditions for 3D culture and protein secretion. The isolated M2 and SMMC7721 cells were resuspended in RPMI 1640 and mixed at a ratio of 1:1, then seeded in 0.75% low-gelling agarose at a concentration of 5 × 10^6^ cells/mL and placed into a standard 6-well culture plate. M2 and SMMC7721 cells were seeded alone in the same conditions as controls. The plates were then incubated at 37 °C for 30 min to allow gelation, after which 1 mL of serum-containing RPMI 1640 was added to each well, followed by incubation at 37 °C in 5% CO_2_ atmosphere for 48 h. The 3D cultures were observed under an inverted phase contrast microscope.

To quantitatively detect the secreted protein in the 3D culture system, serum-free RPMI 1640 medium was used. After incubation for 48 h, the CM in the 3D M2 and SMMC7721 cell cultures, and M2/SMMC7721 cell co-cultures was collected. Following centrifugation at 4,000 × *g* at 4 °C for 30 min using an Amicon^®^ Pro Affinity Concentration Kit (Merck Millipore, Germany) for protein concentration, the collected CM was immediately frozen at −80 °C until the time of proteomic analysis.

### Sample preparation, iTRAQ labeling, and LC-MS/MS analysis

The three concentrated protein groups were labeled with mass-balanced isobaric tags (iTRAQ^TM^ Reagent Kit, Applied Biosystems, USA) as follows: M2CM-118 isobaric tag, co-culture CM-119 isobaric tag, and SMMC7721 cell CM (SMMC7721CM)-121 isobaric tag. LC-MS/MS analysis was carried out according to a previous study^45^ and summarized in Supplementary Figure S2.

### Quantitative and bioinformatic analysis of secretion protein in the 3D culture

The raw MS/MS analysis data was extracted with ProteinPilot 4.5 and further analyzed using Mascot (Matrix Science) set up to search the human database. For iTRAQ quantification, Scaffold (version Scaffold 4.3.2, Proteome Software Inc., Portland, OR, USA) was used to quantitate label-based quantitation peptide and protein identifications. The peptides were identified at the 1% false decoy rate level, and protein identifications were accepted with at least two specific peptides with a minimum 95% confidence. All results were then exported into Excel for further manual data interpretation. To minimize false positive results, a strict cutoff for identification of differentially-expressed proteins was applied, with ratios >1.3 or <0.7 considered up- or down-regulated. The screened-out secretion proteins were further analyzed using the DAVID Bioinformatics Resources 6.7 (http://david.abcc.ncifcrf.gov/). Gene ontology and pathway analyses were performed to reveal the properties of these differential proteins.

### Western blot analysis

Condition medium from different 3D culture systems were used to validate the iTRAQ result. Equal amounts of protein (40 μg) were firstly separated by 10% SDS-PAGE and transferred onto PVDF membrane. After blocking for nonspecific binding (5% nonfat milk in TBS containing Tween20 (TBS-T)) at room temperature for 1 h, the membranes were incubated with Matrix metallopeptidase 3 (MMP3), IL-8, IL-1ß, Chemokine (C-X-C motif) ligand 2 (CXCL2), heparin-binding EGF-like growth factor (HBEGF), or insulin-like growth factor binding protein 3 (IGFBP3) overnight at 4 °C. Followed an extensively washed with TBS-T, the membranes were then incubated with HRP-conjugated secondary antibodies for 1 h at room temperature. After being washed in TBS-T three times, protein bands were finally visualized using chemiluminescence detection and quantified by QUANTITY ONE software. To confirm equal protein loading, Coomassie staining was used.

CXCL2 was further validated in clinical samples using the same method. Tissue samples (including HCC tissue and corresponding non-tumor normal tissues) from 10 HCC patients were collected from the Department of Hepatobiliary Surgery, First Affiliated Hospital of Guangxi Medical University (Nanning, China). General information of these patients was present in Table 1. All patients were HBV positive but negative for antibodies against hepatitis C virus and hepatitis D virus, and all HCC were diagnosed by ultrasound imaging and biopsy. The experiments were carried out according to the principles expressed in the Declaration of Helsinki. And this study was approved by the Medical Ethics Committee of First Affiliated Hospital of Guangxi Medical University, with written informed consent obtained from each participant.

### Effect of different CXCL2 doses on HCC cells

SMMC7721 cells were seeded in 6-well tissue culture dishes at a density of 10^5^ cells/well. Following 24 h of incubation, cells were treated with 0, 1, 10 and 100 ng/mL of CXCL2 in serum-free DMEM for further 24 h. CXCL2 was reconstituted in sterile ultrapure H_2_O and added into the serum-free DMEM to its final concentration.

#### Cell migration and invasion assays

Following treatment with different does of CXCL2 for 24 h, the migration and invasive capability of SMMC7721 cells was assessed as mentioned above.

#### Cell adhesion assay

Fibronectin (FN) at 10 μg/mL concentration was added to the 96-well plates, at a volume of 50 μL/well. The 96-well plates were then placed in the clean bench for seasoning overnight. The plates were then washed with PBS to remove the undesired FN, and 2 × 10^4^ CXCL2-induced SMMC7721 cells were seeded in each well with three replication holes. Following incubation for 30, 60 and 90 min at 37 °C in 5% CO_2_, the non-adherent cells were washed away, and the remaining adherent cells were fixed, stained, and counted under the microscope.

#### Detection of metastasis-related genes

Considering the possible changes of metastasis-related genes during the treatment of CXCL2, the relative expression of two common markers of epithelial-mesenchymal transition (EMT), E-cadherin and N-cadherin, were detected using qRT-PCR and normalized to β-actin. Primers of these genes are presented in Table 2. The reactions were carried out using an IQ5 Multicolor Real-time PCR Detection System with the following amplification conditions: 95 °C for 5 min, 40 cycles of 95 °C for 15 s, 60 °C for 15 s, and 72 °C for 20 s. After amplification, the relative expression value of the PCR products was calculated by 2^−△△CT^ method.

### CXCL2 Neutralization

To further confirm the effect of CXCL2 on SMMC7721 cells, cell migration and invasion assays were conducted again, using the treated condition medium from the co-culture system with its containing CXCL2 neutralized by anti-CXCL2 antibody (Abcam, UK). The co-culture CM was firstly collected and incubated with 1 ug/mL and 10 ug/mL anti-CXCL2 antibody for 2 h at 37 °C, respectively. SMMC7721 cells, after trypsinized and resuspended in FBS-free DMEM, were then plated in the upper chamber and inserted in a well of a 24-well plate containing 300 μL serum-containing medium (10%) mixed with 300 μL of the different treated co-culture CM. Cells inserted in a well with 300 ul untreated co-culture CM as control. A normal control with 300 ul serum-containing medium (10%) and 300 μL serum-free medium was also included.

### CXCR2 inhibition

Since CXCL2 belongs to CXC chemokines family, and binds specifically to CXC chemokine receptor 2 (CXCR2) to play roles in various biological process^46^, further evaluation on the function of CXCL2 was conducted by inhibiting the activity of its receptor CXCR2 with a specific antagonist SB225002(Selleck Chemicals, USA). The concentration of SB225002 (0.1 ug/mL and 1 ug/mL) was chosen according to Wang *et al.*^47^ who found that blocking CXCR2 with 400 nmol/L (~0.13 ug/mL) SB225002 could significantly decreased cell proliferation. Our 48 hours’ proliferation assay also showed that following 24 hours’ treatment with SB225002, proliferation ability of SMMC7721 cells decreased by ~40% to 50%, in both concentration, no matter the present or absent of CXCL2. However, after 48 hours’ treatment, the inhibition effect of cell proliferation became slow and decreased only ~10%–20% (see supplementary Figure S3). Hence, to inhibit the activity of CXCR2, SMMC7721 cells were firstly plated at 10^5^ per well in a six-well plate and treated with 0.1 ug/mL and 1 ug/mL SB225002 in serum-free DMEM for 2 h; then 10 ng/mL CXCL2 was added into the medium and incubated for a further 24. Cells treated with 10 ng/mL CXCL2 were treated as control, a blank control incubated with neither CXCL2 nor SB225002 was also conducted. Finally, cell migration, invasion and adhesion assays were performed using the different treated SMMC7721 cells, as mentioned above.

### Statistical analysis

The data are expressed as mean ± SD. A commercially available statistical software package (SPSS for Windows, 16.0) was used to perform the statistical analyses. Quantitative variables were analyzed via two-tailed unpaired Student’s t-test and P < 0.05 was considered statistically significant.

## Additional Information

**How to cite this article**: Lu, Y. *et al.* Type conversion of secretomes in a 3D TAM2 and HCC cell co-culture system and functional importance of CXCL2 in HCC. *Sci. Rep.* **6**, 24558; doi: 10.1038/srep24558 (2016).

## Supplementary Material

Supplementary Information

## Figures and Tables

**Table 1:** General information of HCC patients.

Feature	HCC
Number of individuals	10
Gender (male/female)	7/3
Age (years, mean ± SD)	52 ± 11
HBV DNA (copy)	2.3 × 10^4^ (1.3 × 10^3^ ~ 1.7 × 10^5^)
AFP (ng/mL)	12354.6(3.2~70321)
HbsAg^+^ (%)	100
AST (U/L)	130.4 (16 ~ 1630)
ALT (U/L)	110.3 (14 ~ 1120)

**Table 2:** List of primers used for qRT-PCR.

Gene	Primer sequences
TNF-α	Forward: 5′-TAGCCCATGTTGTAGCAAACC-3′; Reverse: 5′-ATGAGGTACAGGCCCTCTGAT-3′
CCL3	Forward: 5′-GCTGACTACTTTGAGACGAGCA-3′; Reverse: 5′-ATATTTCTGGACCCACTCCTCA-3′
AMAC-1	Forward: 5′- GCTGCCTCGTCTATACCTCCT-3′; Reverse: 5′-GGTCGCTGATGTATTTCTGGA-3′
CCL18	Forward: 5′-TACCTCCTGGCAGATTCCAC-3′; Reverse: 5′-CCCACTTCTTATTGGGGTCA-3′
CCL22	Forward: 5′-TGATTACGATCCGTTACCGTCT-3′; Reverse: 5′-CCTGAAGGTTAGCAACACCAC -3′
Arg-1	Forward: 5′- CCCTTTGCTGACATCCCTAAT-3′; Reverse: 5′-GGCTGATTCTTCCGTTCTTCT-3′
E-cadherin	Forward :5′-GAATGACAACAAGCCCGAAT-3′; Reverse:5′-GACCTCCATCACAGAGGTTCC-3′
N-cadherin	Fo rward :5′-GGTGGAGGAGAAGAAGACCAG-3′ ; Reverse:5′-GCATCAGGCTCCACAGT-3′
Vimentin	Forward: 5′-TCCGCACATTCGAGCAAAGA-3′; Reverse: 5′-TGAGGGCTCCTAGCGGTTTA-3′
a-SMA	Forward: 5′-CTTGTCCAGGAGTTCCGCTC-3′; Reverse: 5′- TTTCTTGGGCCTTGATGCGA-3′
β-actin	Forward: 5′-CATGTACGTTGCTATCCAGGC -3′; Reverse: 5′- CTCCTTAATGTCACGCACGAT-3′
