# Supplementary material for: Type conversion of secretomes in a 3D TAM2 and HCC cell co-culture system and functional importance of CXCL2 in HCC
Source: Sci Rep. 2016 Apr 27;6:24558. doi: 10.1038/srep24558 (PMC4846822; doi:10.1038/srep24558)
Supplement: Supplementary Information [file srep24558-s1.pdf]

Type conversion of secretomes in a 3D TAM2 and HCC cell co-culture system and functional importance of CXCL2 in HCC

Yu Lu[1]*, Shan Li[1]*, Liping Ma[1]*, Xiaolian Zhang[1], Qiliu Peng[1], Cuiju Mo[1], Li Huang[1], Xue Qin[1#], Yinkun Liu[2, 3#]

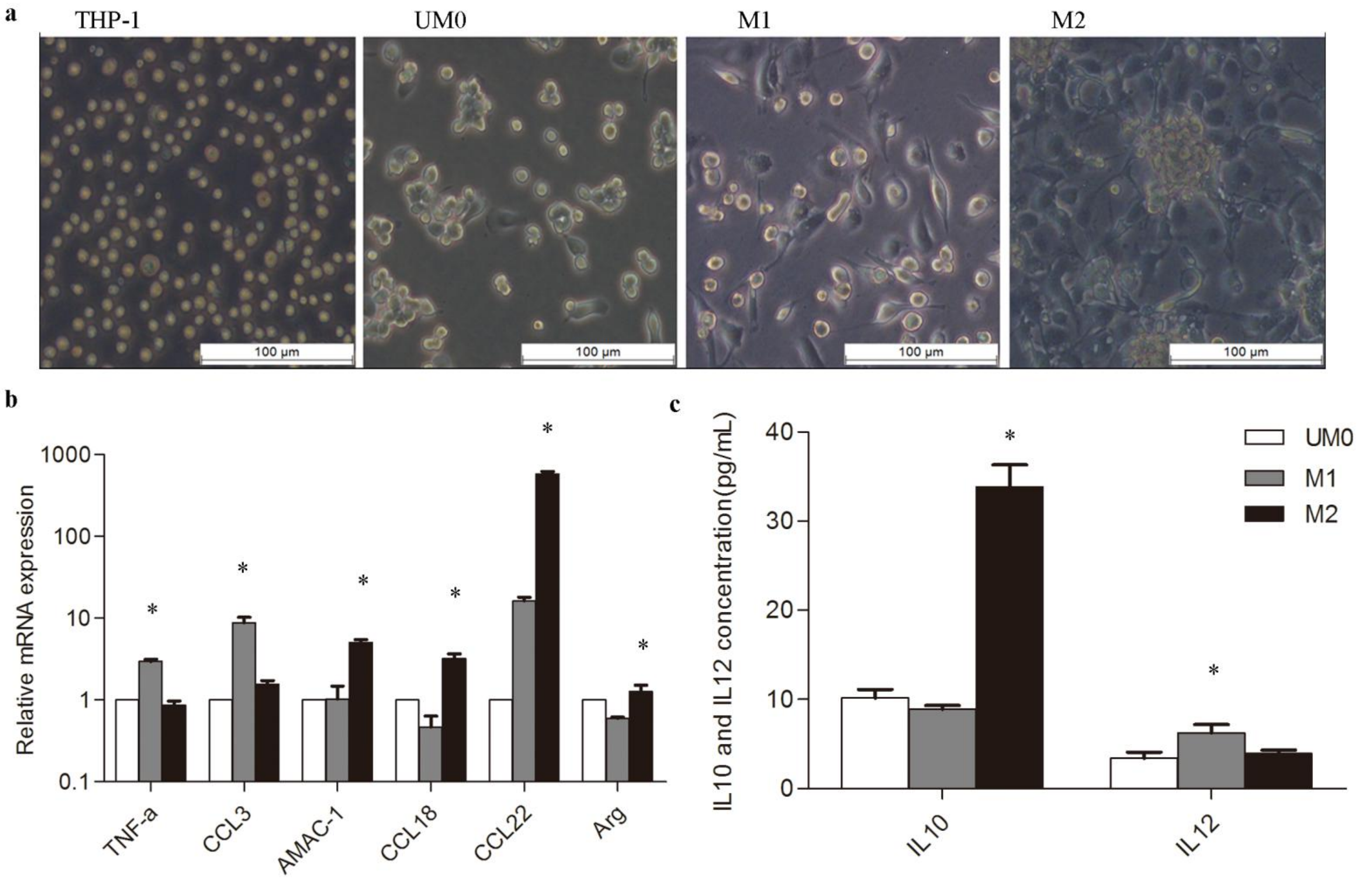

Figure S1 Characteristics of different macrophage phenotypes. a The morphology of undifferentiated macrophages (UM0), classically activated macrophage (M1) and alternatively activated macrophage (M2). The human monocytic cell line THP-1 has been widely used as a model for monocyte/macrophage differentiation. After treatment with PMA for 24 h, round-shaped THP-1 cells quickly stopped proliferating, became attached, and were induced into UM0. The addition of LPS to UM0 lead to elongated, fibroblast-like shaped cells, whereas the stimulation by IL-4 and IL-13 did not induce visible changes in macrophage morphology but led to a longer and disorganized pseudopodium. b The mRNA expression of common markers in UM0, M1 and M2. Tumor necrosis factor-alpha (TNF-α) and chemokine (C-C motif) ligand (CCL) 3 are characteristic markers for M1, while M2 is characterized by its high expression of alternative macrophage activation-associated CC-chemokine (AMAC)-1, CCL18, CCL22, and arginase (Arg)-1. c Cytokine concentration in conditioned medium of UM0, M1 and M2. IL-12^{high} IL-10^{low} represents the M1 phenotype and IL-12^{low} IL-10^{high} the M2 phenotype. The results showed a low level of IL-10 (8.87 ± 0.46 pg/mL) but high levels

of IL-12 (33.82 ±2.50 pg/mL) in M1CM, and a high level of IL-10 (6.25 ±0.92 pg/mL) but low level of IL-12 (2.50 ±0.40 pg/mL) in M2CM. * p<0.05 (all compared to the control group using Student's t-test).

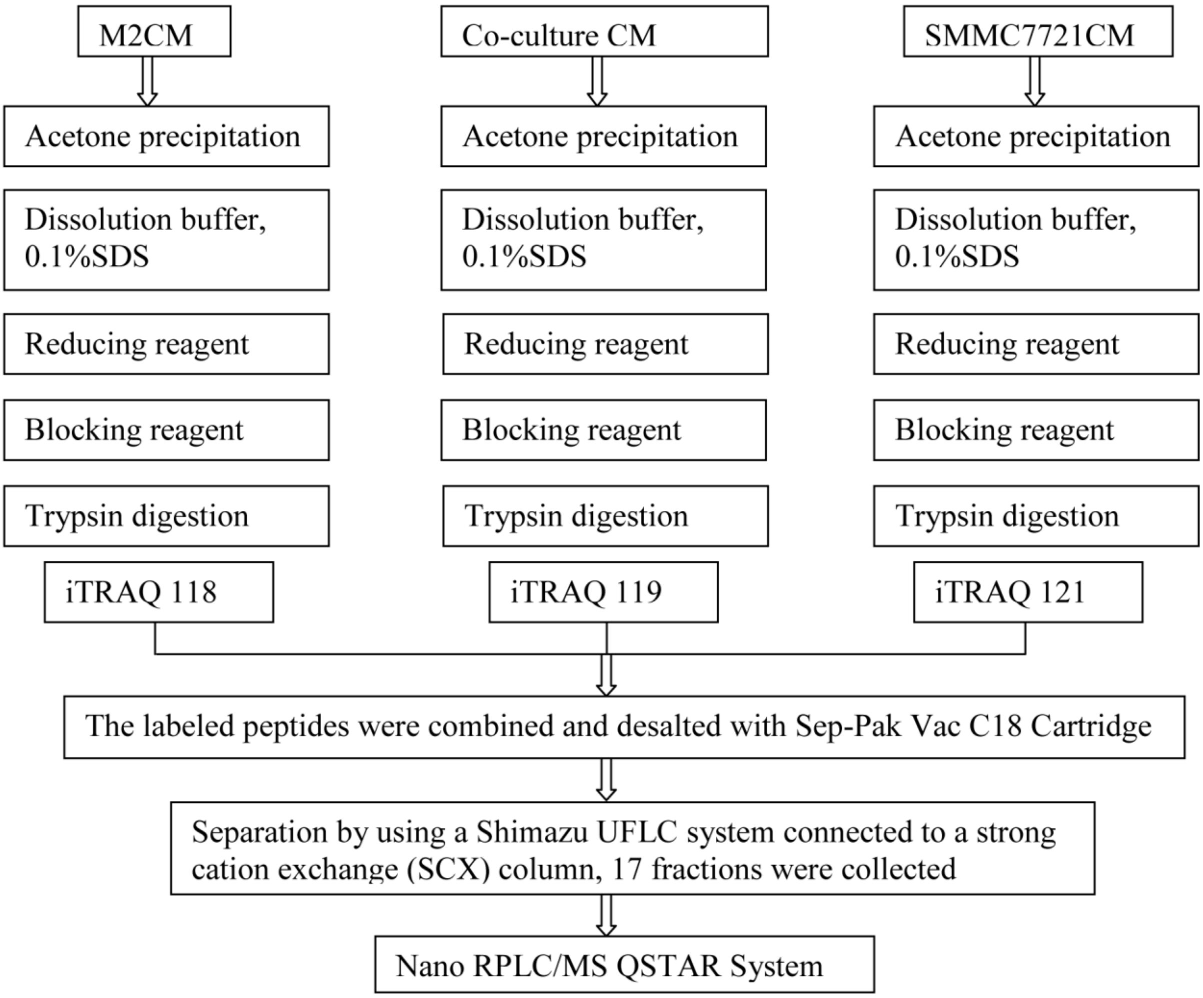

Figure S2 The flow chart of iTRAQ analysis.

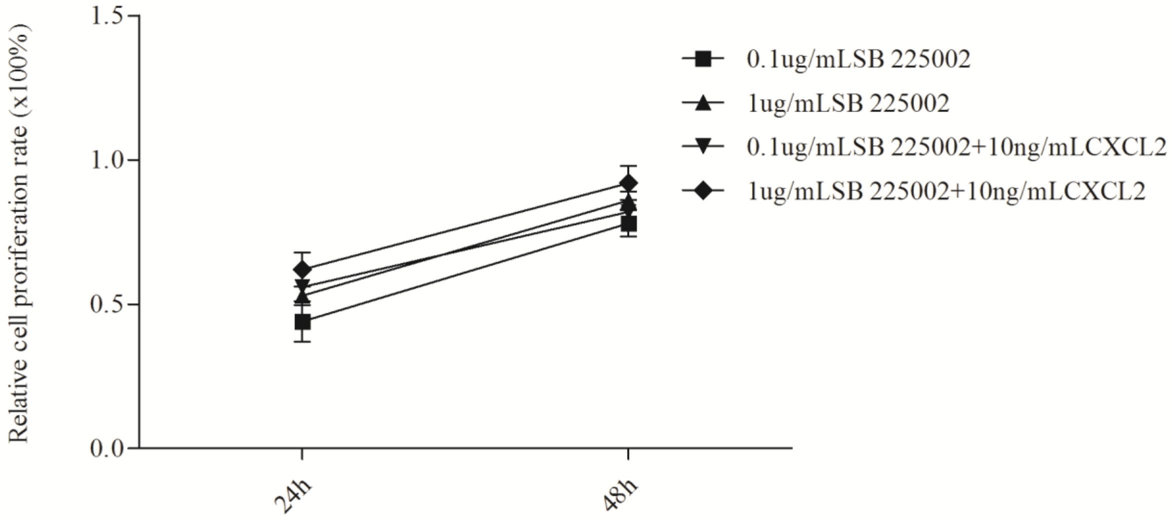

Figure S3 Proliferation assay of SMMC7721 after treated with different does of SB225002. Following 24 hours' treatment with SB225002, proliferation ability of SMMC7721 cells decreased by ~ 40% to 50%, in both 0.1ug/mL and 1ug/mL concentrations, no matter the present or absent of CXCL2. However, after 48 hours' treatment, the inhibition effect of cell proliferation became slow and decreased only ~10%-20%.

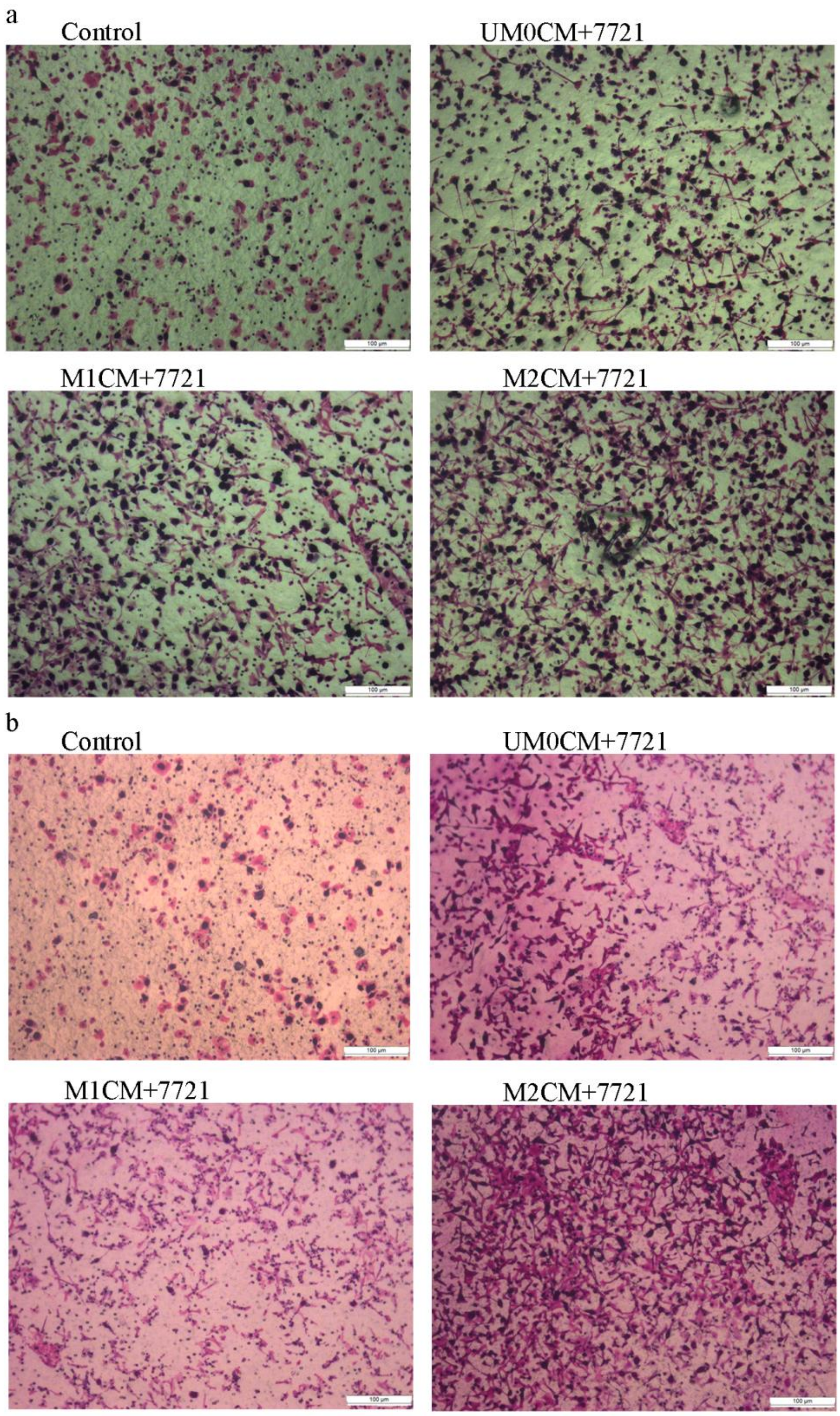

Figure S4 The morphology change of SMMC7721cells during the migration and matrigel invasive assay. They were the larger fields of view of Figure 2a-b.

Table S1 Up-regulated proteins in 3D co-culture system.

Accession no.	Description	119:118	119:121	Family
**IL8_HUMAN**	**interleukin 8**	**3.48**	**6.96**	**cytokine**
TNFA_HUMAN	tumor necrosis factor	2.14	4.92	cytokine
RHOC_HUMAN	ras homolog family member C	2.14	4.59	enzyme
SDC2_HUMAN	syndecan 2	2.63	3.73	other
TFPI2_HUMAN	tissue factor pathway inhibitor 2	2.00	3.48	other
**CXCL2_HUMAN**	**chemokine (C-X-C motif) ligand 2**	**2.00**	**1.51**	**cytokine**
CCL3_HUMAN	chemokine (C-C motif) ligand 3	2.00	3.03	cytokine
APOC2_HUMAN	apolipoprotein C-II	2.00	3.03	transporter
CADH9_HUMAN	cadherin 9, type 2 (T1-cadherin)	2.29	3.03	other
STC1_HUMAN	stanniocalcin 1	4.92	3.03	other
SLAF7_HUMAN	SLAM family member 7	1.31	2.83	other
UPAR_HUMAN	plasminogen activator, urokinase receptor	1.86	2.83	transmembrane receptor
LDLR_HUMAN	low density lipoprotein receptor	2.14	2.83	transporter
SRGN_HUMAN	serglycin	2.29	2.83	other
INHBA_HUMAN	inhibin, beta A	2.46	2.83	growth factor
E41L2_HUMAN	erythrocyte membrane protein band 4.1-like 2	2.46	2.64	other
AMPN_HUMAN	alanyl (membrane) aminopeptidase	1.41	2.46	peptidase
LIF_HUMAN	leukemia inhibitory factor	1.62	2.46	cytokine
VASP_HUMAN	vasodilator-stimulated phosphoprotein	1.74	2.46	other
PI51C_HUMAN	phosphatidylinositol-4-phosphate 5-kinase, type I, gamma	2.00	2.46	kinase
AT2B1_HUMAN	ATPase, Ca++ transporting, plasma membrane 1	3.03	2.46	transporter
HOME3_HUMAN	homer homolog 3 (Drosophila)	1.74	2.29	other
OSTP_HUMAN	secreted phosphoprotein 1	1.41	2.14	cytokine
CD44_HUMAN	CD44 molecule (Indian blood group)	1.62	2.14	enzyme
LIRB2_HUMAN	leukocyte immunoglobulin-like receptor, subfamily B (with TM and ITIM)	2.14	2.14	other
APOE_HUMAN	apolipoprotein E	1.31	2.00	transporter
**IL1B_HUMAN**	**interleukin 1, beta**	**1.86**	2.00	**cytokine**
E41L3_HUMAN	erythrocyte membrane protein band 4.1-like 3	2.29	2.00	other
CD82_HUMAN	CD82 molecule	1.51	1.86	other
CXCL5_HUMAN	chemokine (C-X-C motif) ligand 5	1.51	1.86	cytokine
GLSK_HUMAN	glutaminase	2.29	1.86	ion channel
ICLN_HUMAN	chloride channel, nucleotide-sensitive, 1A	2.46	1.86	peptidase
TXLNA_HUMAN	taxilin alpha	3.48	1.86	cytokine
C1QT1_HUMAN	C1q and tumor necrosis factor related protein 1	11.31	1.86	other
OR1M1_HUMAN	olfactory receptor, family 1, subfamily M, member 1	1.31	0.8	G-protein coupled receptor
ADAM9_HUMAN	ADAM metallopeptidase domain 9	1.62	0.8	peptidase
GROA_HUMAN	chemokine (C-X-C motif) ligand 1 (melanoma growth stimulating activity	1.74	0.8	cytokine
TR19L_HUMAN	RELT tumor necrosis factor receptor	4.00	0.8	transmembrane receptor
**HBEGF_HUMAN**	**heparin-binding EGF-like growth factor**	**1.41**	**3.03**	**growth factor**

ADEC1_HUMAN	ADAM-like, decysin 1	1.62	1.62	peptidase
EHD4_HUMAN	EH-domain containing 4	1.74	1.62	enzyme
PAI1_HUMAN	serpin peptidase inhibitor, clade E (nexin, plasminogen activator inhibitor	2.14	1.62	other
ITA5_HUMAN	integrin, alpha 5 (fibronectin receptor, alpha polypeptide)	1.31	1.51	transmembrane receptor
VPS54_HUMAN	vacuolar protein sorting 54 homolog (S. cerevisiae)	1.41	1.51	other
**IBP3_HUMAN**	**insulin-like growth factor binding protein 3**	**1.51**	**1.62**	**other**
1B15_HUMAN	major histocompatibility complex, class I, B	2.14	1.51	transmembrane receptor
BMP1_HUMAN	bone morphogenetic protein 1	1.31	1.41	peptidase
BASI_HUMAN	basigin (Ok blood group)	1.62	1.41	transporter
S4A7_HUMAN	solute carrier family 4, sodium bicarbonate cotransporter, member 7	1.62	1.41	transporter
VGF_HUMAN	VGF nerve growth factor inducible	1.62	1.41	growth factor
BIN2_HUMAN	bridging integrator 2	1.74	1.41	other
VAT1_HUMAN	vesicle amine transport 1	1.31	1.31	transporter
FA96A_HUMAN	family with sequence similarity 96, member A	1.31	1.31	other
ICAM1_HUMAN	intercellular adhesion molecule 1	1.41	1.31	transmembrane receptor
SAP_HUMAN	prosaposin	1.41	1.31	other
LIPB1_HUMAN	PTPRF interacting protein, binding protein 1 (liprin beta 1)	1.41	1.31	other
**MMP3_HUMAN**	**matrix metallopeptidase 3 (stromelysin 1, progelatinase)**	**1.41**	**1.31**	**peptidase**
TJAP1_HUMAN	tight junction associated protein 1 (peripheral)	1.41	1.31	other
GRAP1_HUMAN	GRIP1 associated protein 1	1.41	1.31	other
XIRP1_HUMAN	xin actin-binding repeat containing 1	1.41	1.31	other
UTRO_HUMAN	utrophin	1.41	1.31	transmembrane receptor
NKTR_HUMAN	natural killer-tumor recognition sequence	1.41	1.31	other
ANKY2_HUMAN	ankyrin repeat and MYND domain containing 2	1.41	1.31	other

118, 118 isobaric tag, labeled the conditioned medium of M2; 119, 119 isobaric tag, labeled the conditioned medium of co-culture; 121, 121 isobaric tag, labeled the conditioned medium of SMMC7721 cells. 119:118, the fold change of proteins expressed in co-culture system when compared with the M2 culture system; 119:121, the fold change of proteins expressed in co-culture system when compared with the SMMC7721 culture system. Those in black were chosen for further verification.

Table S2 Down-regulated proteins in 3D co-culture system.

Accession no.	Description	119:118	119:121	Family
HTRA1_HUMAN	HtrA serine peptidase 1	0.29	0.10	peptidase
FZD6_HUMAN	frizzled family receptor 6	0.54	0.14	G-protein coupled receptor

FETUA_HUMAN	alpha-2-HS-glycoprotein	0.47	0.19	other
NGAL_HUMAN	Neutrophil gelatinase-associated lipocalin	0.57	0.22	transporter
ICAM5_HUMAN	intercellular adhesion molecule 5, telencephalin	0.62	0.22	other
LOXL2_HUMAN	lysyl oxidase-like 2	0.66	0.25	enzyme
AAAT_HUMAN	solute carrier family 1 (neutral amino acid transporter), member 5	0.22	0.27	transporter
T2R60_HUMAN	taste receptor, type 2, member 60	0.47	0.27	G-protein coupled receptor
GRASP_HUMAN	GRP1 (general receptor for phosphoinositides 1)-associated scaffold protein	0.66	0.27	other
O7E24_HUMAN	olfactory receptor, family 7, subfamily E, member 24	0.62	0.29	G-protein coupled receptor
C1QT6_HUMAN	C1q and tumor necrosis factor related protein 6	0.66	0.29	other
ALBU_HUMAN	albumin	0.33	0.31	transporter
THBG_HUMAN	s Thyroxine-binding globulin	0.35	0.31	transporter
PRTN3_HUMAN	proteinase 3	0.35	0.31	peptidase
DDR1_HUMAN	discoidin domain receptor tyrosine kinase 1	0.76	0.31	kinase
TSP4_HUMAN	thrombospondin 4	0.35	0.35	other
PCDA3_HUMAN	protocadherin alpha 3	0.41	0.35	other
AFAM_HUMAN	afamin	0.50	0.35	transporter
PCDH7_HUMAN	protocadherin 7	0.54	0.35	other
USH1C_HUMAN	Usher syndrome 1C (autosomal recessive, severe)	0.33	0.38	other
MIF_HUMAN	macrophage migration inhibitory factor (glycosylation-inhibiting factor)	0.44	0.41	cytokine
CFAD_HUMAN	complement factor D (adipsin)	0.62	0.41	peptidase
RNAS4_HUMAN	ribonuclease, RNase A family, 4	0.62	0.41	enzyme
REEP6_HUMAN	receptor accessory protein 6	0.27	0.44	other
PLXA1_HUMAN	plexin A1	0.31	0.44	transmembrane receptor
PCDA1_HUMAN	protocadherin alpha 1	0.44	0.44	other
PVRL2_HUMAN	poliovirus receptor-related 2 (herpesvirus entry mediator B)	0.47	0.44	transmembrane receptor
PTPRM_HUMAN	protein tyrosine phosphatase, receptor type, M	0.54	0.44	phosphatase
NOTC2_HUMAN	notch 2	0.54	0.44	transcription regulator
CO4A2_HUMAN	collagen, type IV, alpha 2	0.62	0.44	other
1A02_HUMAN	major histocompatibility complex, class I, A	0.18	0.47	other
CBPM_HUMAN	carboxypeptidase M	0.29	0.47	peptidase
CHSTF_HUMAN	carbohydrate (N-acetylgalactosamine 4-sulfate 6-O) sulfotransferase 15	0.44	0.47	enzyme
CBPD_HUMAN	carboxypeptidase D	0.50	0. 47	peptidase
MRC2_HUMAN	mannose receptor, C type 2	0.66	0.47	transmembrane receptor
RAP2A_HUMAN	RAP2A, member of RAS oncogene family	0.47	0.50	enzyme
FETA_HUMAN	alpha-fetoprotein	0.50	0.50	transporter
PTPRF_HUMAN	protein tyrosine phosphatase, receptor type, F	0.62	0.50	phosphatase
USP9X_HUMAN	ubiquitin specific peptidase 9, X-linked	0.62	0.50	peptidase
MMP19_HUMAN	matrix metallopeptidase 19	0.57	0.54	peptidase
IDE_HUMAN	insulin-degrading enzyme	0.66	0.54	peptidase

KCAB2_HUMAN	potassium voltage-gated channel, shaker-related subfamily, beta member 2	0.62	0.57	ion channel
TIE1_HUMAN	tyrosine kinase with immunoglobulin-like and EGF-like domains 1	0.62	0.57	kinase
MIME_HUMAN	Mimecan	0.66	0.57	growth factor
VLDLR_HUMAN	very low density lipoprotein receptor	0.57	0.62	transporter
SCRN2_HUMAN	secernin 2	0.66	0.62	other
ANGT_HUMAN	angiotensinogen (serpin peptidase inhibitor, clade A, member 8)	0.50	0.66	growth factor
MMP14_HUMAN	matrix metallopeptidase 14 (membrane-inserted)	0.66	0.66	peptidase
CXD3_HUMAN	gap junction protein, delta 3	2.83	0.14	transporter
SDC1_HUMAN	syndecan 1	2.00	0.19	enzyme
L1CAM_HUMAN	L1 cell adhesion molecule	1.74	0.23	other
FBLN1_HUMAN	Fibulin-1	2.14	0.25	other
PPB1_HUMAN	alkaline phosphatase, placental	3.25	0.27	phosphatase
MUC18_HUMAN	melanoma cell adhesion molecule	1.62	0.31	other
SMOC1_HUMAN	SPARC related modular calcium binding 1	2.00	0.31	other
VAPB_HUMAN	VAMP (vesicle-associated membrane protein)-associated protein B and C	2.46	0.31	other
LAMB1_HUMAN	laminin, beta 1	1.74	0.35	other
BCAM_HUMAN	basal cell adhesion molecule (Lutheran blood group)	1.74	0.35	transmembrane receptor
DSG2_HUMAN	desmoglein 2	1.32	0.38	other
LAMA4_HUMAN	laminin, alpha 4	1.52	0.38	enzyme
VAPA_HUMAN	VAMP (vesicle-associated membrane protein)-associated protein A	1.32	0.41	other
APOA1_HUMAN	apolipoprotein A-I	1.32	0.41	transporter
CO7A1_HUMAN	collagen, type VII, alpha 1	1.52	0.41	other
FBN1_HUMAN	fibrillin 1	1.41	0.44	other
SCAPE_HUMAN	S-phase cyclin A-associated protein in the ER	1.41	0.44	other
PRG4_HUMAN	proteoglycan 4	1.41	0.50	other
C1S_HUMAN	complement component 1, s subcomponent	1.52	0.50	peptidase
TRFE_HUMAN	transferrin	1.74	0.50	transporter
LG3BP_HUMAN	lectin, galactoside-binding, soluble, 3 binding protein	1.87	0.50	transmembrane receptor
AT2B4_HUMAN	ATPase, Ca++ transporting, plasma membrane 4	1.87	0.50	transporter
TFPI1_HUMAN	tissue factor pathway inhibitor (lipoprotein-associated coagulation inhibitor)	1.87	0.50	other
PCDH1_HUMAN	protocadherin 1	1.87	0.54	other
PGS2_HUMAN	decorin	1.62	0.57	other
FIBA_HUMAN	fibrinogen alpha chain	1.62	0.57	other
TRFM_HUMAN	antigen p97 (melanoma associated) identified by monoclonal antibodies 133.2 and 96.5	1.62	0.57	other
CYR61_HUMAN	cysteine-rich, angiogenic inducer, 61	1.74	0.57	other
LAMC1_HUMAN	laminin, gamma 1 (formerly LAMB2)	1.87	0.57	other
FSTL1_HUMAN	follistatin-like 1	4.92	0.57	other
IGHA1_HUMAN	immunoglobulin heavy constant alpha 1	1.41	0.62	other
CBPA4_HUMAN	carboxypeptidase A4	1.74	0.62	peptidase

IBP7_HUMAN	insulin-like growth factor binding protein 7	2.00	0.62	transporter
MAGD2_HUMAN	melanoma antigen family D, 2	1.41	0.66	other
DAF_HUMAN	CD55 molecule, decay accelerating factor for complement (Cromer blood group)	1.41	0.66	other
SSFA2_HUMAN	sperm specific antigen 2	1.62	0.66	other
CO1A1_HUMAN	collagen, type I, alpha 1	1.87	0.66	other
CH3L1_HUMAN	chitinase 3-like 1 (cartilage glycoprotein-39)	0.66	1.32	enzyme
A2MG_HUMAN	alpha-2-macroglobulin	0.62	1.41	transporter
CLC11_HUMAN	C-type lectin domain family 11, member A	0.66	1.41	growth factor
SEM7A_HUMAN	semaphorin 7A, GPI membrane anchor (John Milton Hagen blood group)	0.57	1.62	transmembrane receptor
MMP9_HUMAN	matrix metallopeptidase 9 (gelatinase B, 92kDa gelatinase, 92kDa type IV collagenase)	0.50	1.62	peptidase
CSF1R_HUMAN	colony stimulating factor 1 receptor	0.57	1.62	kinase
KLRF1_HUMAN	killer cell lectin-like receptor subfamily F, member 1	0.57	1.74	transmembrane receptor
REEP5_HUMAN	receptor accessory protein 5	0.23	1.87	transporter
TIMP1_HUMAN	TIMP metallopeptidase inhibitor 1	0.62	1.87	other
CAP1_HUMAN	CAP, adenylate cyclase-associated protein 1 (yeast)	0.54	2.46	other
PNOC_HUMAN	prepronociceptin	0.62	3.25	other

118, 118 isobaric tag, labeled the conditioned medium of M2; 119, 119 isobaric tag, labeled the conditioned medium of co-culture; 121=121 isobaric tag, labeled the conditioned medium of SMMC7721 cells. 119:118, the fold change of proteins expressed in co-culture system when compared with the M2 culture system; 119:121, the fold change of proteins expressed in co-culture system when compared with the SMMC7721 culture system.
